# Mg^2+^ supplementation treats secretory diarrhea in mice by activating calcium-sensing receptor in intestinal epithelial cells

**DOI:** 10.1172/JCI171249

**Published:** 2024-01-16

**Authors:** Livia de Souza Goncalves, Tifany Chu, Riya Master, Parth D. Chhetri, Qi Gao, Onur Cil

**Affiliations:** Department of Pediatrics, UCSF, San Francisco, California, USA.

**Keywords:** Cell Biology, Calcium, Chloride channels, Epithelial transport of ions and water

## Abstract

Cholera is a global health problem with no targeted therapies. The Ca^2+^-sensing receptor (CaSR) is a regulator of intestinal ion transport and a therapeutic target for diarrhea, and Ca^2+^ is considered its main agonist. We found that increasing extracellular Ca^2+^ had a minimal effect on forskolin-induced Cl^–^ secretion in human intestinal epithelial T84 cells. However, extracellular Mg^2+^, an often-neglected CaSR agonist, suppressed forskolin-induced Cl^–^ secretion in T84 cells by 65% at physiological levels seen in stool (10 mM). The effect of Mg^2+^ occurred via the CaSR/Gq signaling that led to cAMP hydrolysis. Mg^2+^ (10 mM) also suppressed Cl- secretion induced by cholera toxin, heat-stable *E*. *coli* enterotoxin, and vasoactive intestinal peptide by 50%. In mouse intestinal closed loops, luminal Mg^2+^ treatment (20 mM) inhibited cholera toxin–induced fluid accumulation by 40%. In a mouse intestinal perfusion model of cholera, addition of 10 mM Mg^2+^ to the perfusate reversed net fluid transport from secretion to absorption. These results suggest that Mg^2+^ is the key CaSR activator in mouse and human intestinal epithelia at physiological levels in stool. Since stool Mg^2+^ concentrations in patients with cholera are essentially zero, oral Mg^2+^ supplementation, alone or in an oral rehydration solution, could be a potential therapy for cholera and other cyclic nucleotide–mediated secretory diarrheas.

## Abstract 2

Extracellular Mg2+ regulates CaSR activity and inhibits CFTR-mediated Cl- secretion in human intestinal epithelial cells and mouse intestine.

## Graphical abstract

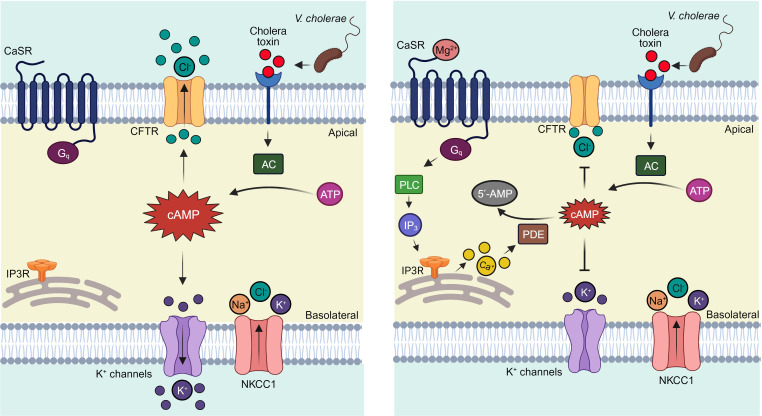

## Introduction

Diarrhea remains an important cause of global mortality, and diarrheal illness accounts for more than 25% of deaths in children under 5 years of age in sub-Saharan Africa and South Asia (1–3). Secretory diarrhea, a major form of diarrhea with diverse etiologies, leads to the activation of prosecretory Cl^–^ channels (such as cystic fibrosis transmembrane conductance regulator [CFTR]) and/or inhibition of proabsorptive ion transporters (such as sodium-hydrogen exchanger 3 [NHE3]) in the luminal membrane of the intestinal epithelial cells (4). Cholera is a severe form of secretory diarrhea, in which the key pathology is increased CFTR-mediated Cl^–^ secretion and reduced NHE3-mediated Na^+^ absorption due to elevated cAMP levels in intestinal epithelial cells (5–10). Current treatment of secretory diarrhea is primarily supportive, with fluid and electrolyte replacement by intravenous fluids or oral rehydration solution (ORS). Commonly used glucose-based ORS formulations rely on intact sodium-glucose cotransporter activity in cholera for electrolyte repletion (11, 12). However, ORS has no effect on stool output, which is considered a key contributor to the low ORS use rates globally (13). Current ORS formulations do not directly address the hypersecretion, and cholera remains an important cause of mortality despite the availability of ORS (14). An improved ORS formulation with antisecretory properties, in addition to fluid and electrolyte repletion, could potentially improve clinical outcomes for patients with cholera or other secretory diarrheas.

The extracellular Ca^2+^-sensing receptor (CaSR) is expressed in many tissues including parathyroid, kidney, bone, brain, and gut (15). In the intestine, the CaSR is a regulator of fluid and electrolyte transport (16, 17). We recently showed that the FDA-approved CaSR activator cinacalcet inhibits CFTR-mediated fluid secretion and promotes NHE3-mediated fluid absorption in human colonic epithelial T84 cells and mouse intestine by promoting cAMP hydrolysis through phosphodiesterases (PDEs). Consistent with this mechanism, cinacalcet was very effective in various mouse models of secretory diarrhea (18, 19). Although CaSR activation is a promising treatment strategy for secretory diarrheas, and short-term cinacalcet use for cholera might provide marked clinical benefits, long-term cinacalcet use for chronic diarrheas can cause systemic side effects due to CaSR activation in other organs. CaSR is physiologically activated by organic cations, including Ca^2+^, which the receptor is named after (20). We hypothesized that CaSR activation by enteral supplementation of its endogenous agonists can be used as a simple, safe, and effective treatment for secretory diarrhea. Although Ca^2+^ is considered the major CaSR agonist, we found here that the extracellular Ca^2+^ concentration had a minimal effect on CaSR activity in human intestinal epithelial cells and mouse intestine. Interestingly, CaSR activity and cAMP-induced Cl^–^ secretion in human intestinal epithelial cells and mouse intestine strongly correlated with the extracellular concentration of Mg^2+^, an often-neglected CaSR agonist. Although Mg^2+^ causes osmotic diarrhea at high stool concentrations (>100 mM) (21), we found here that Mg^2+^ largely inhibited cyclic nucleotide–induced Cl^–^ secretion at physiological levels (10–20 mM) seen in stool. Considering that stool Mg^2+^ concentrations are essentially zero in patients with cholera (22), we postulate that oral Mg^2+^ supplementation (either alone or in ORS) can be a potential treatment for cholera and other secretory diarrheas.

## Results

### Extracellular Ca^2+^ has minimal effect on CFTR-mediated Cl^–^ secretion in T84 cells.

**Figure 1:**
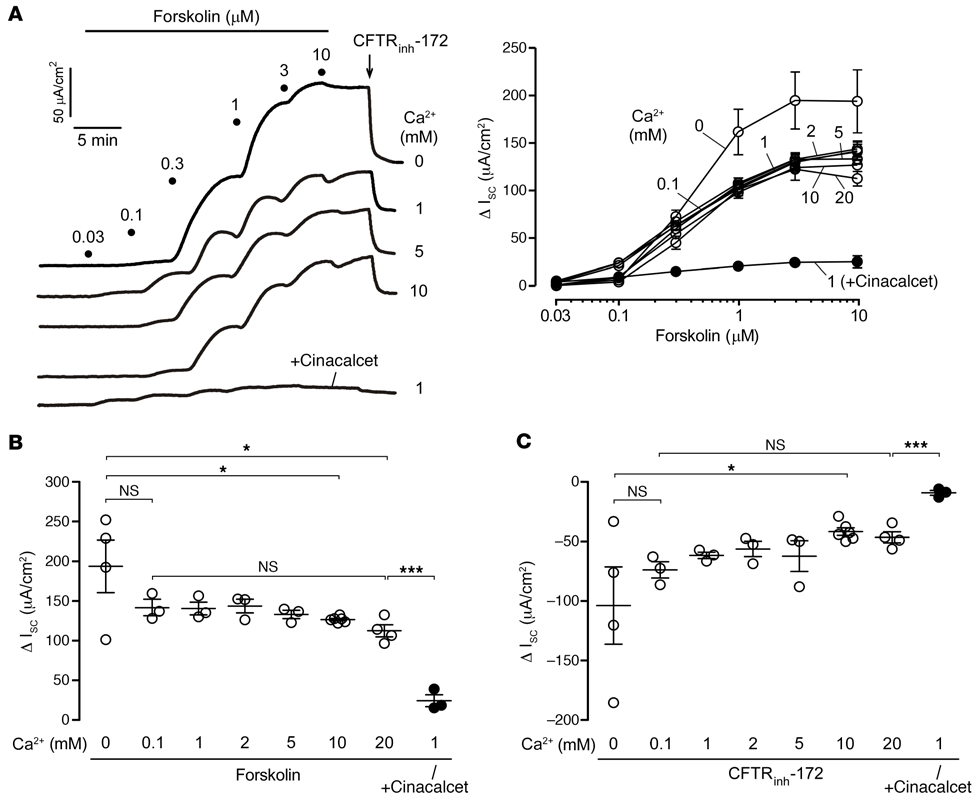
Extracellular Ca^2+^ concentration has minimal effect on forskolin-induced I_sc_ in T84 cells. (**A**) I_sc_ traces in T84 cells showing the forskolin concentration response and CFTR_inh_-172 (10 μM) inhibition following 20 minutes of pretreatment with the indicated concentrations of CaCl_2_ or 30 μM cinacalcet (left). Summary of changes in I_sc_ (Δ I_sc_) from the experiments (right). (**B**) Δ I_sc_ induced by forskolin in the presence of different Ca^2+^ concentrations and cinacalcet. (**C**) Δ I_sc_ induced by CFTR_inh_-172 in the presence of different Ca^2+^ concentrations and cinacalcet. *n* = 3–6 per group. Data indicate the mean ± SEM. *P < 0.05 and ***P < 0.001, by 1-way ANOVA with Newman-Keuls multiple-comparison test.

To test the effect of extracellular Ca^2+^ on Cl^–^ secretion, short-circuit current (I_sc_) measurements were done in human colonic T84 cells bathed with varying concentrations of Ca^2+^. Changing the extracellular Ca^2+^ concentration had minimal effect on forskolin-induced I_sc_, as suggested by similar maximal forskolin responses in the presence of 0.1 to 20 mM Ca^2+^ (Figure 1A). In Ca^2+^-free solution, forskolin caused slightly increased I_sc_ changes compared with 10 mM or higher Ca^2+^ concentrations (Figure 1B). The forskolin-induced secretory current in T84 cells was partially reversed by selective CFTR inhibitor (CFTR_inh_-172) treatment (Figure 1A). CFTR activity in T84 cells was not dependent on the extracellular Ca^2+^ concentration, as indicated by similar CFTR_inh_-172 responses in the presence of 0.1–20 mM Ca^2+^ (Figure 1, A and C). In the same experiments, the CaSR activator drug cinacalcet inhibited forskolin and CFTR_inh_-172–induced I_sc_ changes by 80% (Figure 1, B and C). These results suggest that extracellular Ca^2+^ had a minimal effect on CaSR activity and CFTR-mediated Cl^–^ secretion in human intestinal epithelial cells.

### CFTR-mediated Cl^–^ secretion in T84 cells is strictly dependent on the extracellular Mg^2+^ concentration.

**Figure 2:**
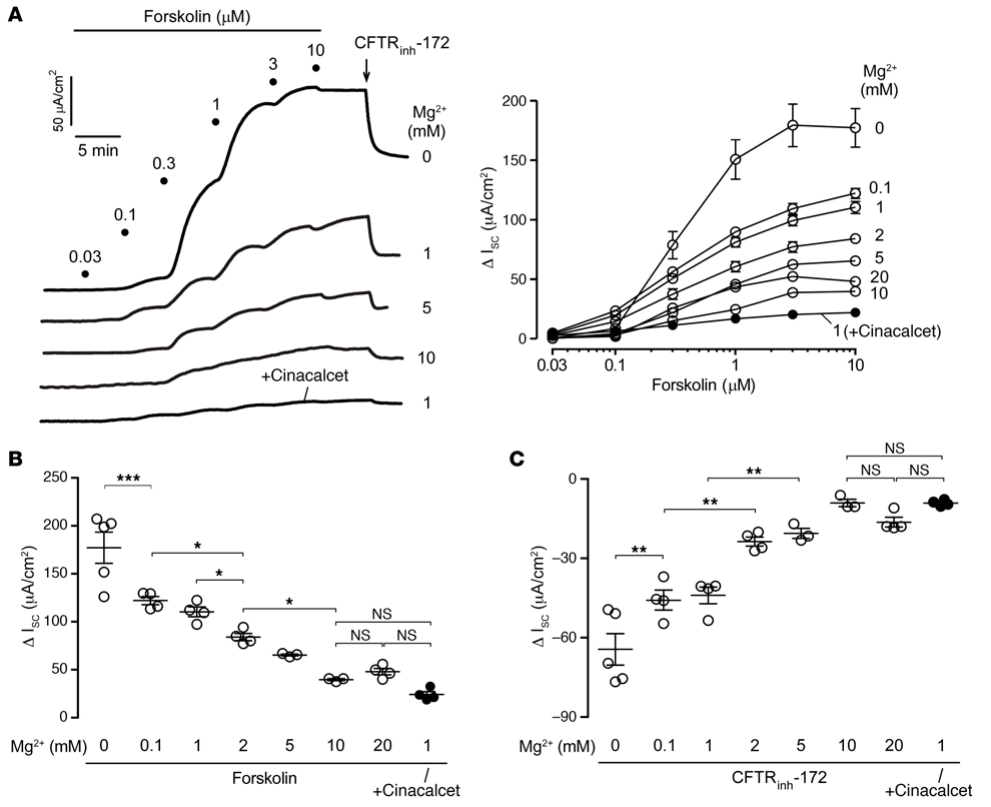
Forskolin-induced I_sc_ in T84 cells is dependent on the extracellular Mg^2+^ concentration. (**A**) I_sc_ traces in T84 cells showing the forskolin concentration response and CFTR_inh_-172 (10 μM) inhibition following 20 minutes of pretreatment with the indicated concentrations of MgCl_2_ or 30 μM cinacalcet (left). Summary of Δ I_sc_ from the experiments (right). (**B**) Δ I_sc_ induced by forskolin in the presence of different Mg^2+^ concentrations and cinacalcet. (**C**) Δ I_sc_ induced by CFTR_inh_-172 at in the presence of different Mg^2+^ concentrations and cinacalcet. *n* = 3–5 per group. Data indicate the mean ± SEM. *P < 0.05, **P < 0.01, and ***P < 0.001, by 1-way ANOVA with Newman-Keuls multiple-comparison test.

Since Ca^2+^ had minimal effect on CaSR activity and Cl^–^ secretion in T84 cells, we next investigated the effects of Mg^2+^, a less-studied physiological CaSR agonist. Forskolin-induced I_sc_ in T84 cells inversely correlated with the extracellular Mg^2+^ (as MgCl_2_) concentration (Figure 2A). Mg^2+^ inhibited forskolin-induced maximal I_sc_ by 70% at 10 mM or higher concentrations (Figure 2B), which is the physiological Mg^2+^ concentration in human stool (21). The antisecretory effect of Mg^2+^ was due to CFTR inhibition, as suggested by substantially lower I_sc_ responses to CFTR_inh_-172 with increasing Mg^2+^ concentrations (Figure 2, A and C). The antisecretory effects of 10 mM or higher Mg^2+^ were comparable to the effect of the CaSR activator cinacalcet, as indicated by the similar forskolin and CFTR_inh_-172 responses. Altering extracellular Ca^2+^ and Mg^2+^ concentrations simultaneously had inhibitory effects on forskolin (Supplemental Figure 1, A and B; supplemental material available online with this article; https://doi.org/10.1172/JCI171249DS1) and CFTR_inh_-172 responses (Supplemental Figure 1, A and C), similar to that seen with alteration of Mg^2+^ alone. Mg^2+^ also had concentration-dependent inhibitory effects on forskolin and CFTR_inh_-172–induced I_sc_ changes when citrate (MgC_6_H_6_O_7_, Supplemental Figure 2, A and B) or sulfate (MgSO_4_, Supplemental Figure 2, C and D) salts of Mg^2+^ were used. Increasing solution Mg^2+^ in these experiments slightly increased the solution osmolality (<10% for 10 mM MgCl_2_ vs. 1 mM MgCl_2_). Although equal osmolality increases by CaCl_2_ did not have antisecretory effects (Figure 1), we performed control studies to directly rule out any potential effects of increased solution osmolality on secretory currents in T84 cells. Addition of 30 or 60 mM mannitol to the solutions (equivalent to adding 10 or 20 mM MgCl_2_, respectively) did not affect forskolin or CFTR_inh_-172 responses, whereas 10 mM Mg^2+^ had marked antisecretory effects in side-by-side studies (Supplemental Figure 3). To rule out any potential effects of Mg^2+^ on barrier permeability, we measured transepithelial electrical resistance (TEER) in the presence of various Mg^2+^ and Ca^2+^ concentrations. Altering extracellular Mg^2+^ or Ca^2+^ concentrations had no effect on TEER under the I_sc_ study conditions (Supplemental Figure 4). These results suggest that extracellular Mg^2+^ is the major CaSR agonist and regulator of CFTR-mediated Cl^–^ secretion in human intestinal epithelial cells.

### Extracellular Mg^2+^ exerts its antisecretory effect by indirect inhibition of CFTR through CaSR activation.

**Figure 3:**
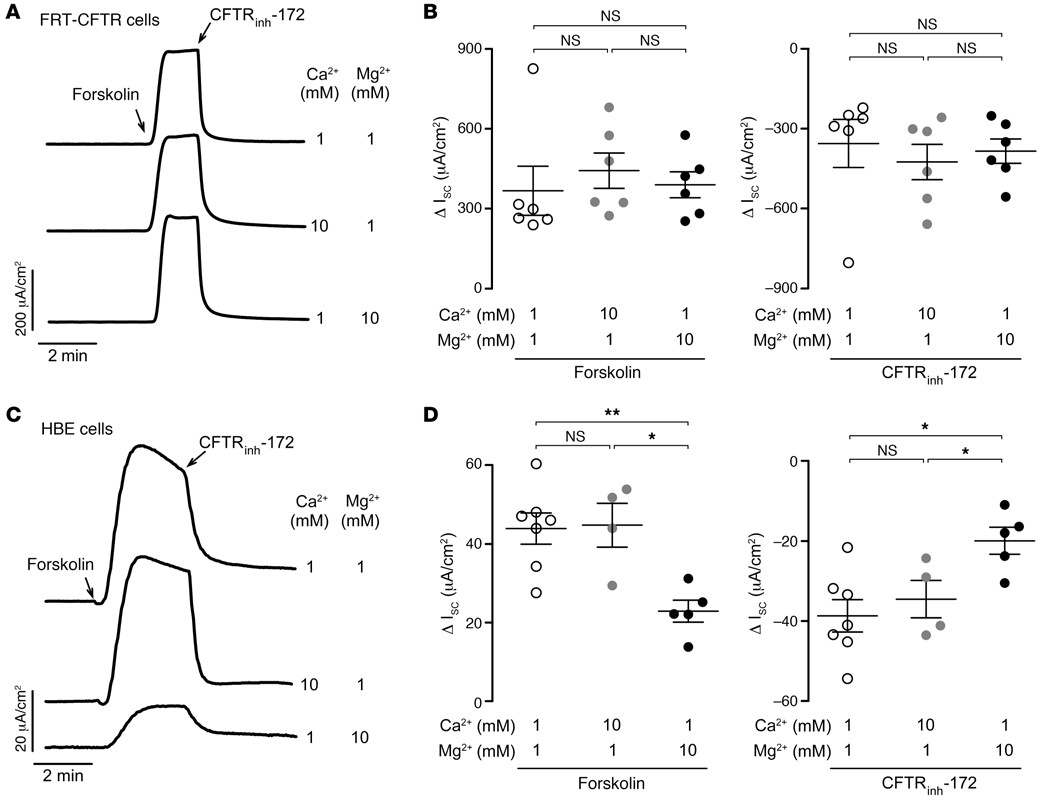
Mg^2+^ inhibition of CFTR-mediated Cl^–^ secretion is indirect and depends on CaSR activation. (**A**) I_sc_ traces in FRT-CFTR cells showing responses to maximal forskolin (10 μM) and CFTR_inh_-172 (10 μM) following 20 minutes of pretreatment with the indicated concentrations of CaCl_2_ or MgCl_2_. (**B**) Summary of Δ I_sc_ induced by forskolin (left) and CFTR_inh_-172 (right) at different Ca^2+^ and Mg^2+^ concentrations. (**C**) I_sc_ traces in HBE cells showing responses to maximal forskolin (10 μM) and CFTR_inh_-172 (10 μM) following 20 minutes of pretreatment with the indicated concentrations of Ca^2+^ or Mg^2+^. (**D**) Δ I_sc_ induced by forskolin (left) and CFTR_inh_-172 (right) at different Ca^2+^ and Mg^2+^ concentrations. *n* = 4–7 per group. Data indicate the mean ± SEM. *P < 0.05 and **P < 0.01, by 1-way ANOVA with Newman-Keuls multiple-comparison test.

To determine whether Mg^2+^ or Ca^2+^ has direct CFTR inhibitory effects, we performed I_sc_ studies in CFTR-transfected Fischer rat thyroid (FRT-CFTR) cells, which do not express the CaSR (23, 24) and are commonly used to study CFTR modulators (18, 25–27). With basolateral membrane permeabilization and a 60 mM basolateral-to-apical Cl^–^ gradient, we found that forskolin induced a large Cl^–^ secretory current in FRT-CFTR cells that was completely reversed by CFTR_inh_-172 treatment (Figure 3A). Increasing the extracellular Mg^2+^ (or Ca^2+^) concentration from 1 to 10 mM had no effect on forskolin or CFTR_inh_-172 responses (Figure 3B), suggesting that Mg^2+^ did not have a direct CFTR inhibitory effect.

The effects of Mg^2+^ and Ca^2+^ on CFTR-mediated Cl^–^ secretion were also investigated in well-differentiated human bronchial epithelial (HBE) cells that express both CFTR and the CaSR, and have robust forskolin-induced Cl^–^ secretory responses (18). Similar to intestinal epithelial cells, we found that increasing the extracellular Mg^2+^ concentration from 1 to 10 mM inhibited forskolin and CFTR_inh_-172 responses in HBE cells by approximately 50% (Figure 3, C and D). However, increasing the extracellular Ca^2+^ concentration from 1 to 10 mM had no effect on forskolin or CFTR_inh_-172 responses in HBE cells, suggesting that Mg^2+^ is also the key CaSR agonist in airway epithelial cells.

### The antisecretory effect of Mg^2+^ in T84 cells occurs through inhibition of apical membrane Cl^–^ and basolateral membrane K^+^ conductance.

**Figure 4:**
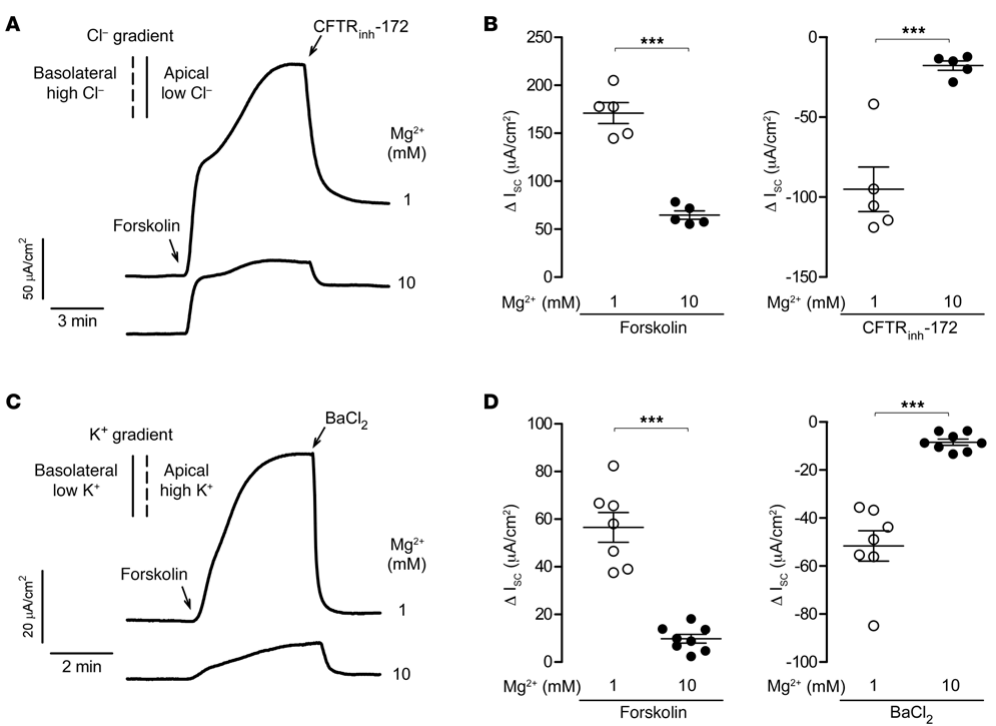
Mg^2+^ inhibits apical membrane CFTR Cl^–^ channel and basolateral membrane K^+^ channels in T84 cells. (**A**) I_sc_ traces in T84 cells with basolateral permeabilization (amphotericin B, 500 μg/mL for 30 min) and a 60 mM basolateral-to-apical Cl^–^ gradient showing responses to 10 μM forskolin and 10 μM CFTR_inh_-172 following 20 minutes of pretreatment with 1 or 10 mM MgCl_2_. (**B**) Summary of Δ I_sc_ induced by forskolin (left) and CFTR_inh_-172 (right) at 1 or 10 mM Mg^2+^. (**C**) I_sc_ traces with apical permeabilization (amphotericin B, 20 μg/mL for 30 min) and an apical-to-basolateral K^+^ gradient showing responses to 10 μM forskolin and 5 mM BaCl_2_ following 20 minutes of pretreatment with 1 or 10 mM Mg^2+^. (**D**) Δ I_sc_ induced by forskolin (left) and BaCl_2_ (right) at 1 or 10 mM Mg^2+^. *n* = 5–8 per group. Data indicate the mean ± SEM. ***P < 0.001, by 2-tailed, unpaired Student’s *t* test.

cAMP-induced secretory I_sc_ in intestinal epithelial cells involves the coordinated action of the apical membrane CFTR Cl^–^ channel and basolateral membrane K^+^ channels (28). To selectively investigate the effect of Mg^2+^ on apical CFTR conductance, we conducted experiments using T84 cells with selective basolateral membrane permeabilization and a basolateral-to-apical Cl^–^ gradient (29, 30). Under these conditions, addition of 10 mM Mg^2+^ to the bathing solution inhibited forskolin and CFTR_inh_-172–induced I_sc_ changes by 70% (Figure 4, A and B). To study the effect of Mg^2+^ on basolateral membrane K^+^ channels, we performed experiments with selective apical membrane permeabilization and an apical-to-basolateral K^+^ gradient (29, 31). In this setting, addition of 10 mM Mg^2+^ to the bathing solution largely reduced basolateral membrane K^+^ conductance, as shown by an 80% reduction in I_sc_ changes in response to forskolin and BaCl_2_ (cAMP-activated K^+^ channel inhibitor) (Figure 4, C and D). These results suggest that Mg^2+^ exerted its antisecretory effect in T84 cells via inhibition of the apical membrane CFTR Cl^–^ channel and basolateral membrane K^+^ channels.

### Mg^2+^ reduces cAMP levels in T84 cells through activation of phospholipase C and PDEs.

**Figure 5:**
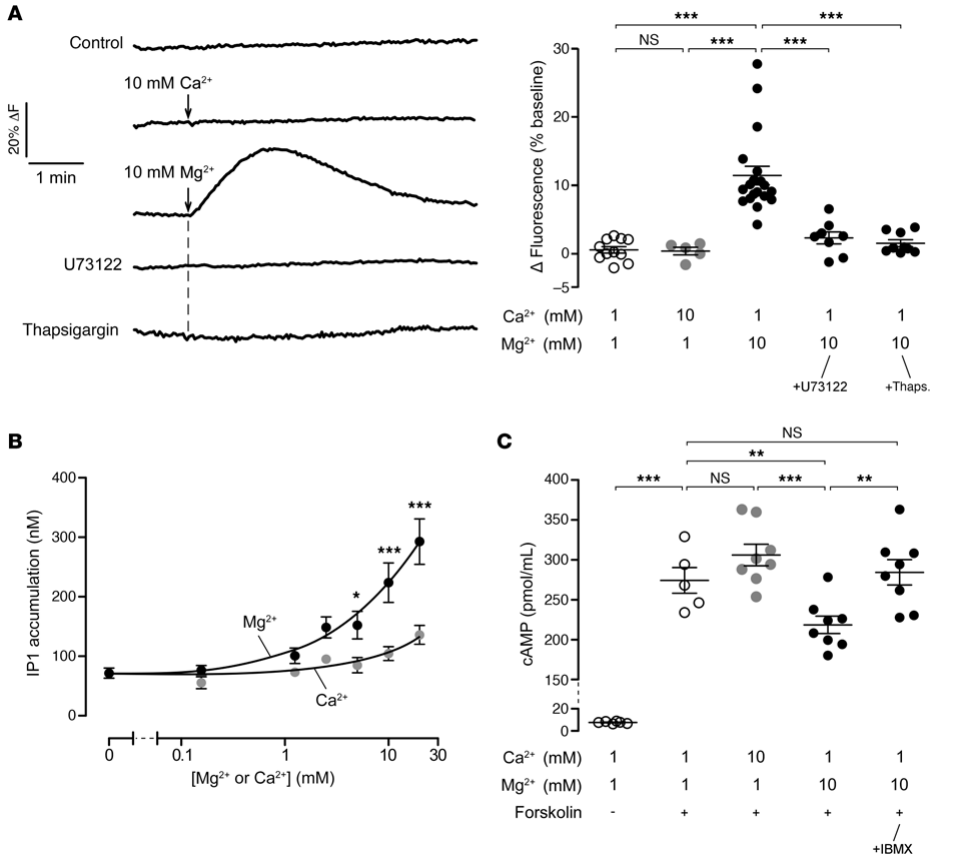
Mg^2+^ increases intracellular Ca^2+^ via Gq and PLC activation and inhibits forskolin-induced cAMP elevation in T84 cells. (**A**) Intracellular Ca^2+^ traces measured by Fluo-4 NW fluorescence in T84 cells with vehicle control (0.1% DMSO), CaCl_2_ (10 mM), or MgCl_2_ (10 mM) (left). In some experiments, T84 cells were pretreated with the PLC inhibitor U73122 (10 μM) or the SERCA inhibitor thapsigargin (1 μM) for 10 minutes before Mg^2+^ addition. Thapsigargin caused a large increase in intracellular Ca^2+^, which is not shown. Summary of data in **A** are presented as maximum changes in Fluo-4 NW fluorescence as a percentage of baseline (right). *n* = 5–20 per group. (**B**) IP1 (stable downstream metabolite of IP3) accumulation after 30 minutes of treatment with 0–20 mM Ca^2+^ or Mg^2+^. *n* = 7–11 per concentration per group. (**C**) cAMP concentration in T84 cell lysates with 10 μM forskolin (± 500 μM IBMX, a phosphodiesterase inhibitor) treatment following 20 minutes of pretreatment with the indicated concentrations of Ca^2+^, Mg^2+^, or vehicle control (0.2% DMSO). *n* = 5–8 per group. Data indicate the mean ± SEM. *P < 0.05, **P < 0.01, and ***P < 0.001, by 1-way ANOVA with Newman-Keuls multiple-comparison test (**A** and **C**) and 2-way ANOVA with Bonferroni’s post test compared with the same concentration of Ca^2+^ (other comparisons were not significant) (**B**).

Activation of phospholipase C (PLC) and consecutive mobilization of intracellular Ca^2+^ by IP3 is the key downstream pathway of CaSR activation, which leads to PDE activation and cAMP hydrolysis in human intestinal epithelial cells (16, 32). To test the role of this mechanism in the Mg^2+^ effect, we measured intracellular Ca^2+^ by Fluo-4 fluorescence. Extracellular addition of 10 mM Mg^2+^ resulted in a marked elevation of intracellular Ca^2+^, which was abolished by pretreatment with the PLC inhibitor U73122 (Figure 5A). Mg^2+^-induced intracellular Ca^2+^ elevation was from intracellular stores, as suggested by the prevention of a Ca^2+^ increase after endoplasmic reticulum stores were depleted by thapsigargin. Consistent with the lack of its antisecretory effects, 10 mM Ca^2+^ had no effect on intracellular Ca^2+^ levels in T84 cells (Figure 5A). IP1 is the stable downstream metabolite of IP3, and its quantification is considered the standard method to assess CaSR activity (33). Similar to the above-cited studies, we observed that extracellular Mg^2+^ concentration-dependently increased intracellular IP1 levels in T84 cells, whereas Ca^2+^ had minimal effect only at high concentrations (Figure 5B). Since cAMP is the major activator of apical CFTR and basolateral K^+^ channels, reduced cAMP levels via CaSR activation might explain the antisecretory effect of Mg^2+^ in T84 cells. Consistent with this mechanism, extracellular addition of 10 mM Mg^2+^ (but not Ca^2+^) reduced the forskolin-induced cAMP elevation in T84 cells (Figure 5C). The effect of Mg^2+^ on cAMP levels was completely reversed with the PDE inhibitor IBMX, pointing to PDE activation as the key mechanism of the Mg^2+^ effect. Collectively, these results further confirmed that Mg^+2^ (but not Ca^2+^) was the major CaSR agonist in human intestinal epithelial cells and that Mg^2+^ exerted its antisecretory effects via the known CaSR signaling pathways including PLC-mediated intracellular Ca^2+^ mobilization and PDE activation.

### Mg^2+^ does not affect Cl^–^ secretion induced by Ca^2+^ agonists.

Although elevation of cAMP — and hence CFTR activation — is the key mechanism in cholera, in certain secretory diarrheas, elevation of intracellular Ca^2+^ is the major driver of Cl^–^ secretion via Ca^2+^-activated Cl^–^ channels (CaCCs). To test the effects of Mg^2+^ on CaCC activity, we performed I_sc_ studies in T84 cells using the cholinergic agonist carbachol. In the presence of 1 or 10 mM Mg^2+^, carbachol induced comparable secretory currents (Supplemental Figure 5), suggesting that CaSR activation by Mg^2+^ did not affect CaCC activity.

### Mg^2+^ inhibits cholera toxin, heat-stable E. coli enterotoxin, and vasoactive intestinal peptide–induced Cl^–^ secretion in T84 cells.

**Figure 6:**
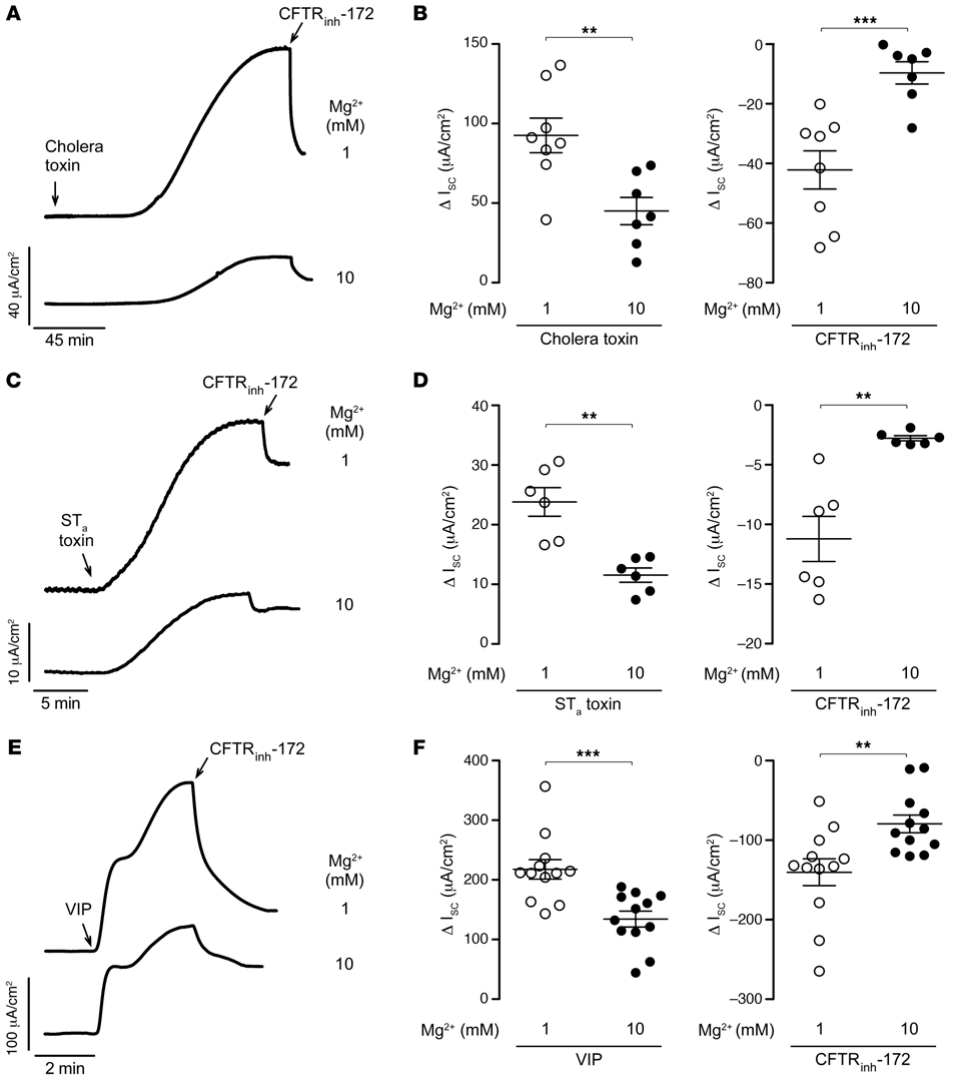
Mg^2+^ inhibits cholera toxin–, ST_a_ toxin–, and VIP-induced Cl^–^ secretion in T84 cells. (**A**) I_sc_ traces showing responses to 1 μg/mL cholera toxin and 10 μM CFTR_inh_-172 with 1 or 10 mM MgCl_2_ pretreatment for 20 minutes. (**B**) Summary of data, as in **A**, showing Δ I_sc_ following cholera toxin (left) and CFTR_inh_-172 (right). (**C**) I_sc_ traces showing responses to 0.1 μg/mL ST_a_ toxin and 10 μM CFTR_inh_-172 with 1 or 10 mM Mg^2+^ pretreatment for 20 minutes. (**D**) Summary of data, as in **C**, showing Δ I_sc_ following ST_a_ toxin (left) and CFTR_inh_-172 (right). (**E**) I_sc_ traces showing responses to 10 nM VIP and 10 μM CFTR_inh_-172 with 1 or 10 mM Mg^2+^ pretreatment for 20 minutes. (**F**) Summary of data, as in **E**, showing Δ I_sc_ following VIP (left) and CFTR_inh_-172 (right). *n* = 6–12 per group. Data indicate the mean ± SEM. **P < 0.01 and ***P < 0.001, by 2-tailed, unpaired Student’s *t* test.

Cyclic nucleotide–mediated (cAMP- or cGMP-mediated) CFTR activation and consequent Cl^–^ secretion represent the key pathology in certain secretory diarrheas including cholera, and traveler’s diarrhea and diarrhea caused by vasoactive intestinal peptide–secreting (VIP-secreting) tumors (VIPomas) (5, 6, 34). To test the efficacy of Mg^2+^ in these settings, we conducted I_sc_ experiments using T84 cells treated with cholera toxin, heat-stable *E. coli* enterotoxin (ST_a_ toxin), and VIP as secretagogues. Increasing extracellular Mg^2+^ concentration from 1 to 10 mM suppressed I_sc_ changes induced by cholera toxin (Figure 6, A and B), ST_a_ toxin (Figure 6, C and D), and VIP (Figure 6, E and F) by greater than 50%. In a similar manner, 10 mM Mg^2+^ treatment resulted in reduced CFTR activity in all experiments, as suggested by markedly lower CFTR_inh_-172 responses compared with controls (Figure 6, B, D, and F, right panels).

### Mg^2+^ has antisecretory effects in mouse intestine via CaSR activation.

**Figure 7:**
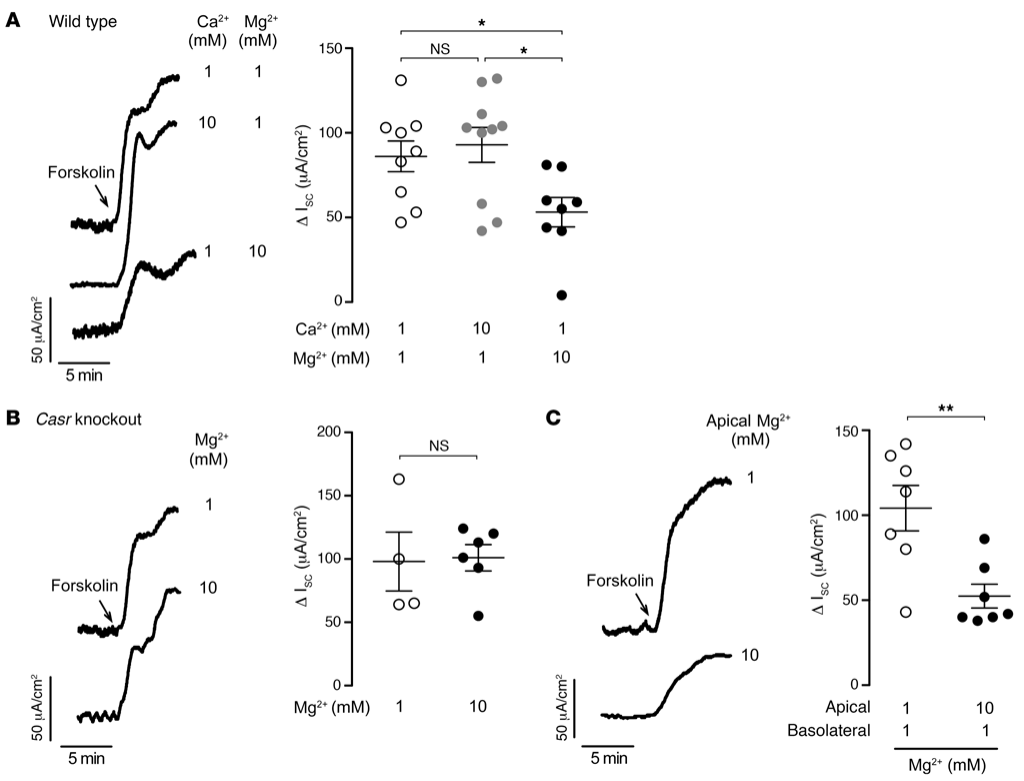
Antisecretory effect of Mg^2+^ in mouse intestine is CaSR dependent. (**A**) I_sc_ traces (left) and summary data (right) showing responses to 10 μM forksolin in WT C57BL/6 mouse jejunum with the indicated concentrations of CaCl_2_ or MgCl_2_ pretreatment from the luminal and basolateral sides for 20 minutes. (**B**) I_sc_ traces (left) and summary data (right) showing responses to 10 μM forksolin in jejunum of intestinal epithelium–specific CaSR-KO mice (*Vil1-Cre Casr ^fl/fl^*) with 1 or 10 mM Mg^2+^ pretreatment from both sides for 20 minutes. (**C**) I_sc_ traces (left) and summary data (right) showing responses to 10 μM forksolin in jejunum of WT mice with 1 or 10 mM Mg^2+^ pretreatment from the luminal (apical) side for 20 minutes. For 10 mM Mg^2+^ experiments, the solution osmolality was balanced between luminal and basolateral solutions by adding 27 mM mannitol to the basolateral side. *n* = 4–10 per group. Data indicate the mean ± SEM. *P < 0.05 and **P < 0.01, by 1-way ANOVA with Newman-Keuls multiple-comparison test (**A**) and 2-tailed, unpaired Student’s *t* test (**B** and **C**).

As done for T84 cells, we tested the antisecretory effect of Mg^2+^ in mouse jejunal mucosa. In WT mice, increasing extracellular Mg^2+^ (but not Ca^2+^) concentration from 1 to 10 mM reduced forskolin-induced secretory I_sc_ by 40% (Figure 7A). Parallel studies were performed in intestinal epithelium–specific CaSR-KO mice (*Vil1-Cre Casr^fl/fl^*), in which 10 mM Mg^2+^ had no antisecretory effects (Figure 7B). These results suggest that CaSR activation was the key mechanism for the antisecretory effect of Mg^2+^ in mouse intestine. Mg^2+^ also had marked antisecretory effects when applied only to the luminal side of the intestine (Figure 7C), which suggests its potential efficacy with oral treatment.

### Efficacy of Mg^2+^ in mouse models of cholera.

**Figure 8:**
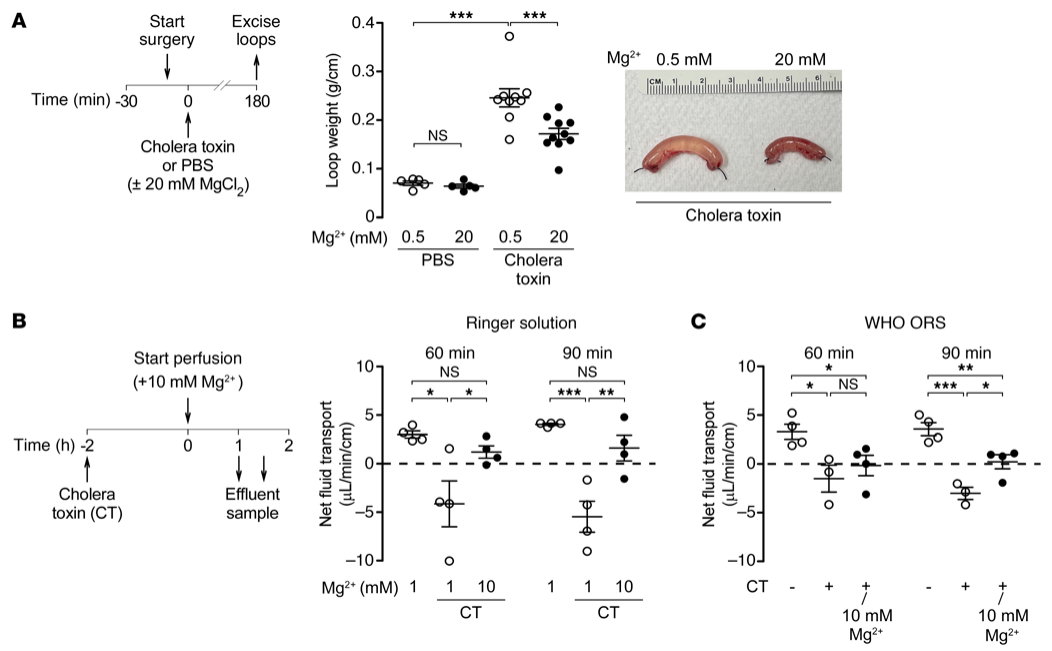
Efficacy of Mg^2+^ in mouse models of cholera. (**A**) Experimental design (left), loop weight/length ratio (center), and representative photos of mouse closed loops (right) injected with 100 μL PBS (± 1 μg cholera toxin) with 0.5 or 20 mM MgCl_2_. *n* = 5–10 loops per group. (**B**) Left: Experimental design of an intestinal perfusion model of cholera in mice induced by intestinal instillation of cholera toxin (10 μg/2 mL saline) or saline control at –2 hours. Right: Intestinal perfusion was done with Ringer’s solution containing 1 or 10 mM Mg^2+^. Net intestinal fluid transport was calculated at the indicated time points. (**C**) Perfusion experiments were done as in **B** using the WHO ORS with and without 10 mM MgCl_2_. *n* = 3–4 mice per group. Data indicate the mean ± SEM. *P < 0.05, **P < 0.01, and ***P < 0.001, by 1-way ANOVA with Newman-Keuls multiple-comparison test.

We tested the efficacy of Mg^2+^ in an intestinal closed-loop mouse model of cholera (Figure 8A, left). In this model, cholera toxin caused marked intestinal fluid accumulation at 3 hours, as suggested by an increased loop weight/length ratio. Intraluminal 20 mM Mg^2+^ treatment at time zero (together with cholera toxin) inhibited the increase in the loop weight/length ratio by 40% (Figure 8A, center and right). We measured the remaining Mg^2+^ concentration in the loops at the end of the 3-hour period and found that the luminal Mg^2+^ concentration dropped to 5.2 ± 1.2 mM, which suggests that the luminal Mg^2+^ concentration might have become slightly subtherapeutic in some loops toward the end of this study. A potential approach to using Mg^2+^ for diarrhea treatment is to fortify the ORS with Mg^2+^, which might provide sustained CaSR activation in the intestine for even higher efficacy. To test this idea, we established a mouse intestinal perfusion model of cholera (Figure 8B, left), in which the Mg^2+^ concentration of the perfusate was controlled. In this model, cholera toxin administration resulted in net intestinal fluid loss, as demonstrated by the negative fluid transport rate. Increasing the Mg^2+^ concentration from 1 to 10 mM reversed net secretion into net absorption, as indicated by positive fluid transport rates (Figure 8B, right). Mg^2+^ (10 mM) also had antisecretory effects in the perfusion model when the WHO ORS solution was used, particularly at 90 minutes, when the cholera toxin effect was fully established (Figure 8C). These results further support our idea of developing a Mg^2+^-fortified ORS for cholera and other secretory diarrheas.

## Discussion

**Figure 9:**
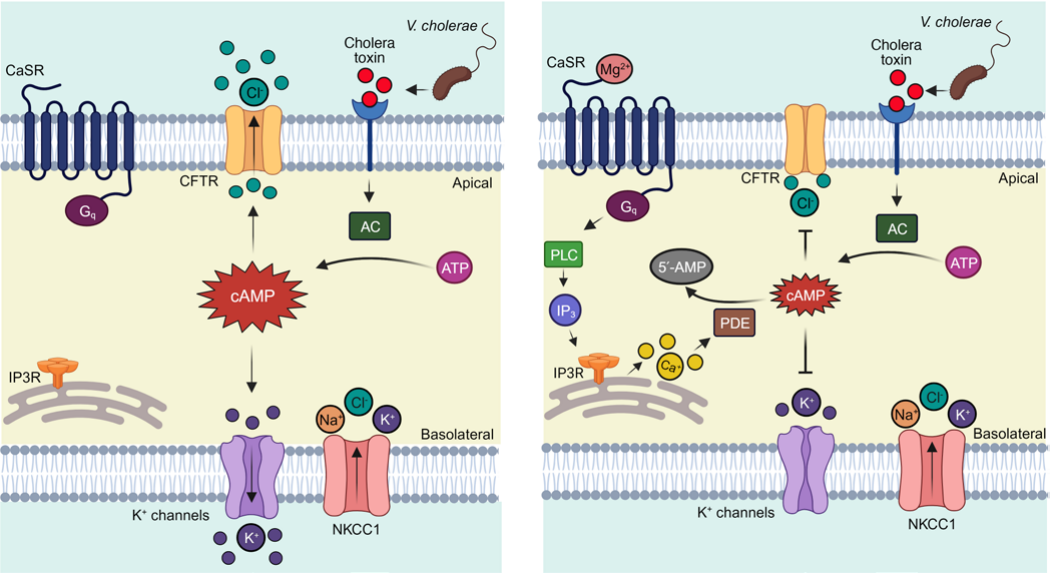
Mechanisms of the effect of Mg^2+^ in secretory diarrheas. Left: Cholera toxin stimulates adenylate cyclase (AC) to elevate cytoplasmic cAMP, which activates the apical membrane CFTR Cl^–^ channel and basolateral membrane K^+^ channels to stimulate Cl^–^ secretion. Right: Mg^2+^ treatment activates CaSR which stimulates the Gq/PLC/IP3 pathway and promotes cAMP hydrolysis by PDE. This process results in reduced activity of CFTR and K^+^ channels and decreases intestinal Cl^–^ secretion. Image was created with Biorender.com.

Here, we showed that CFTR-mediated Cl^–^ secretion in human intestinal epithelial cells and mouse intestine was dependent on extracellular Mg^2+^ concentration, which exerted its effect through CaSR activation (see Figure 9 for the proposed mechanisms). Interestingly, Ca^2+^, which is considered the main physiological CaSR agonist, had minimal effect on CaSR activity and Cl^–^ secretion in intestinal epithelial cells.

The antidiarrheal effect of Mg^2+^ shown here might sound contradictory, since oral Mg^2+^ supplements can cause osmotic diarrhea at high doses (21). The normal range of feces-soluble Mg^2+^ concentration is 10–30 mM in healthy individuals, which increases to 100–150 mM in individuals with Mg^2+^-induced diarrhea (21). Although Mg^2+^ can cause osmotic diarrhea at concentrations of greater than 100 mM, our findings suggest that physiological Mg^2+^ concentrations in the intestinal lumen have antidiarrheal effects through CaSR activation. In patients with cholera and VIP-induced diarrhea, there is a lack of a stool osmotic gap (22, 35), which indirectly suggests the possibility that stool Mg^2+^ concentrations might be low in these conditions. Our findings suggest that increasing stool Mg^2+^ concentrations to physiological levels (10–20 mM) by oral supplementation might offer a simple, safe, and effective therapy for secretory diarrheas. In addition, the stool Mg^2+^ concentration can potentially be implemented as a secretory diarrhea biomarker to identify patients with low stool Mg^2+^ who are likely to benefit from Mg^2+^ supplementation. However, we would like to note that the earlier studies mentioned above did not directly measure stool Mg^2+^ concentrations in patients, and thus the lack of a stool osmotic gap in these studies may also be explained by other factors such as potential measurement errors. Future clinical studies formally quantifying stool Mg^2+^ concentrations in patients with cholera or other forms of diarrhea may be informative to validate its utility as a biomarker.

The current treatment for cholera primarily relies on the ORS that was developed after the discovery of intact glucose-dependent Na^+^ absorption in secretory diarrheas (11, 12). However, the ORS does not have any effects on hypersecretion or stool output (4, 13). Based on our results, Mg^2+^ can be used as an adjunct therapy that can reduce fluid secretion and stool output. One potential issue with oral Mg^2+^ treatment is its relatively rapid intestinal absorption as shown in our closed-loop studies, which may require frequent administration in severe diarrheas such as cholera. Additional dose/frequency determination, pharmacokinetics, and pharmacodynamics studies may be informative prior to testing the efficacy of intermittent oral Mg^2+^ treatment. Alternatively, we postulate that the addition of 10 mM Mg^2+^ to the ORS (Mg^2+^-fortified ORS) can provide sustained CaSR activation in the intestine and reduce stool output, in addition to repleting fluid and electrolytes. Although we present evidence for the efficacy of this approach in an animal model, further preclinical studies are required to optimize the formulation of Mg^2+^-fortified ORS that could ultimately be tested side-by-side with traditional ORS in clinical trials. A theoretical concern with the use of Mg^2+^ in diarrhea treatment is potential hypermagnesemia as a side effect, since Mg^2+^ salts have 50%–67% oral bioavailability (36). However, serum Mg^2+^ levels are tightly regulated by the kidneys, which can rapidly decrease or increase Mg^2+^ excretion according to dietary changes (37, 38). Thus, the absorbed Mg^2+^ is predicted to be rapidly excreted by the kidneys with minimal or no elevation in serum Mg^2+^ levels. Another theoretical concern might be that repeated Mg^2+^ treatment may result in depletion of the IP3-sensitive Ca^2+^ pool in intestinal epithelia. As discussed above, the physiological Mg^2+^ concentration in human stool fluid is 10–30 mM, which potentially suggests constitutive CaSR activation in the intestine under normal conditions. Considering the potentially low Mg^2+^ concentrations in cholera and other secretory diarrheas discussed above, short-term Mg^2+^ treatment may be effective in acute secretory diarrheas by restoring physiological Mg^2+^ concentrations and CaSR activity in the intestine. Future studies testing the efficacy of long-term Mg^2+^ treatment and potential tolerance development in chronic diarrheas may be informative to demonstrate the efficacy of Mg^2+^ treatment in the chronic setting.

Earlier studies investigating the roles of the CaSR in intestinal fluid transport solely focused on Ca^2+^ and indicated Ca^2+^ dependence of forskolin-induced Cl^–^ secretion in rat colonocytes (32, 39). On the basis of these results, the effects of Ca^2+^ supplementation on diarrhea were studied in earlier clinical trials. Oral Ca^2+^ supplementation was shown to have a mild antidiarrheal effect in traveler’s diarrhea, though mainly by preventing bacterial colonization (40). A large randomized, controlled trial in children compared the effects of low-calcium (50 mg/day) and regular-calcium (440 mg/day) milk on the number and duration of diarrhea episodes and found no benefits of higher calcium intake (41). Although the role of the CaSR in diarrhea has been known for decades (4, 42), there are no large-scale clinical studies showing beneficial effects of Ca^2+^ supplementation. Our findings here suggest that Mg^2+^ (but not Ca^2+^) is the key CaSR agonist in intestinal epithelia, which can potentially explain the lack of antisecretory effects of Ca^2+^ in previous clinical studies.

Certain compounds that elevate intracellular Ca^+2^ (such as cholinergic agonists) induce Cl^–^ secretion, which is thought to be mediated by CaCCs. Although CaSR activation by Mg^2+^ also increased intracellular Ca^2+^, Mg^2+^ did not induce a secretory current in T84 cells, similar to what we have previously found with the CaSR-activator drug cinacalcet (18). In addition, Mg^2+^ did not affect cholinergic agonist carbachol-induced secretory currents as shown here. Although cytosolic Ca^+2^ elevation is a shared mechanism between CaSR and cholinergic agonists, additional unshared signaling pathways, including crosstalk with EGF signaling (43, 44), might be important determinants for the secretory effects of cholinergic agonists, but not CaSR agonists.

Certain secretory diarrheas (such as those caused by rotavirus) are characterized primarily by CaCC-mediated Cl^–^ secretion. Here, we found that CaSR activation by Mg^2+^ largely inhibited cyclic nucleotide agonist–induced Cl^–^ secretion, without any effects on Ca^2+^ agonist–induced Cl^–^ secretion. Thus, Mg^2+^ may not be effective in secretory diarrheas in which CaCC activation is the major driver of intestinal fluid loss. Although our results here showed a marked inhibitory effect of Mg^2+^ on CFTR-mediated Cl^–^ secretion, CaCCs may also play a role in intestinal fluid loss in cyclic nucleotide–mediated diarrheas. In addition, there can be crosstalk between cAMP and Ca^2+^ pathways, which can lead to activation of both CFTR and CaCCs in certain secretory diarrheas (4). Future studies investigating the effects of Mg^2+^ on different secretory pathways and crosstalk mechanisms may be lead to a better understanding of its mechanisms of action and potential efficacy in patients with diarrhea.

Although we showed here that Mg^2+^ was effective in both cAMP- and cGMP-mediated diarrhea models, cyclic nucleotide elevation is not a common pathology in all diarrheas. Thus, Mg^2+^ (alone or in an ORS) may not be effective as a general antidiarrheal, but it can potentially be used as a specific and targeted treatment for cyclic nucleotide–mediated diarrheas such as cholera and traveler’s diarrhea and diarrhea induced by VIPoma and *GUCY2C* mutations.

The majority of earlier studies on CaSR agonists used bovine parathyroid cells or HEK-293 cells transfected with CaSR (45). In both settings, Ca^2+^ is 2- to 3-fold more potent CaSR agonist than Mg^2+^ (46–48). Consistent with this, the serum ionized Ca^2+^ concentration is the primary determinant of parathyroid hormone (PTH) secretion in vivo (49). We show here that in human intestinal and airway epithelial cells natively expressing the CaSR, Mg^2+^ was the key agonist for this receptor. These results also suggest that the term “calcium-sensing receptor” might be an oversimplification of the biological roles of this receptor.

In conclusion, we have demonstrated that the extracellular Mg^2+^ concentration was the major regulator of CaSR activity and cAMP-induced Cl^–^ secretion in intestinal epithelial cells. Oral Mg^2+^ supplementation, either alone or in an ORS, can offer a simple, safe, targeted and effective treatment for cyclic nucleotide–mediated secretory diarrheas such as cholera and traveler’s diarrhea or those induced by VIPoma and *GUCY2C* mutations.

## Methods

### Cell culture.

T84 cells (ATCC CCL–248, human colon carcinoma cells) were cultured as previously described (18, 29) on inserts (12 mm diameter, 0.4 μm pore size; Corning Life Sciences) and used for I_sc_ experiments 7 days after plating. FRT cells stably expressing human WT CFTR (FRT-CFTR cells) were cultured as described previously (50) on inserts and used for I_sc_ experiments 5 days after plating. Well-differentiated HBE cells were cultured at an air-liquid interface on inserts as described before (51). HBE cells were used for I_sc_ experiments 21 days after plating, when they typically form a tight epithelium (TEER >1,000 Ω cm^2^).

### I_sc_ measurements.

Cells were mounted in Ussing chambers containing bicarbonate-buffered Ringer’s solution at pH 7.4 (120 mM NaCl, 5 mM KCl, 1 mM CaCl_2_, 1 mM MgCl_2_, 10 mM D-glucose, 5 mM HEPES, 25 mM NaHCO_3_). The MgCl_2_ and/or CaCl_2_ concentrations of both apical and basolateral solutions were altered (0–20 mM) in separate experiments as indicated in each figure. Secretagogues and ion channel inhibitors were added to both apical and basolateral bathing solutions. In some experiments, to measure apical Cl^–^ conductance, the basolateral membrane was permeabilized with 500 μg/mL amphotericin B for 30 minutes and a 60 mM basolateral-to-apical Cl^–^ gradient was applied. For these experiments, Ringer’s was the basolateral bathing solution (120 mM NaCl), and the apical solution contained 60 mM NaCl and 60 mM sodium gluconate. To measure basolateral membrane K^+^ conductance, the apical membrane was permeabilized with 20 μM amphotericin B for 30 minutes, and an apical-to-basolateral potassium gradient was applied. The apical solution (pH 7.4) contained 142.5 mM K-gluconate, 1 mM CaCl_2_, 1 mM MgCl_2_, 0.43 mM KH_2_PO_4_, 0.35 mM Na_2_HPO_4_, 10 mM HEPES, and 10 mM D-glucose. In the basolateral solution (pH 7.4), 142.5 mM K-gluconate was replaced with 5.5 mM K-gluconate and 137 mM *N*-methylglucamine. The solutions were aerated with 95% O_2_/5% CO_2_ and maintained at 37°C during the experiments. The I_sc_ was measured using an EVC4000 multichannel voltage clamp (World Precision Instruments) via Ag/AgCl electrodes and 3 M KCl agar bridges. In parallel experiments, T84 cells were grown on permeable filters as described above and bathed with Ringer’s solution containing 0–10 mM CaCl_2_ and/or MgCl_2_ for 60 minutes. TEER was measured using a Millicell-ERS Resistance System with a dual-electrode volt-ohmmeter (MilliporeSigma) to test the effects of various Ca^2+^ and Mg^2+^ concentrations on epithelial barrier function. Net TEER (Ω/cm^2^) was calculated by subtracting the resistance of cell-free media from the measured resistance (52).

### Chemicals.

All chemicals were purchased from MilliporeSigma except CFTR_inh_-172 (MedChemExpress) and ST_a_ toxin (Bachem Americas).

### CaSR activity and cAMP measurements.

For intracellular Ca^2+^ measurements, T84 cells were plated in 96-well, black-walled microplates (Corning). Confluent cells were loaded with the Ca^2+^ indicator Fluo-4 NW (Invitrogen) according to the manufacturer’s instructions. Fluo-4 fluorescence was measured in each well continuously with a Tecan Infinite M1000 plate reader (Tecan Group) at excitation/emission wavelengths of 495 nm/516 nm after addition of 10 mM CaCl_2_ or MgCl_2_. In some experiments, cells were pretreated with the PLC inhibitor U73122 (10 μM) or the sarcoplasmic/endoplasmic reticulum Ca^2+^-ATPase (SERCA) inhibitor thapsigargin (1 μM) for 10 minutes prior to addition of MgCl_2_. For the IP1 assay, T84 cells were grown in 384-well opaque plates (PerkinElmer) and treated with 0–20 mM CaCl_2_ or MgCl_2_ for 30 minutes. Next, cells were lysed and the IP1 concentration in each well was quantified using the IP-One Gq kit (Cisbio) according to the manufacturer’s instructions. For the cAMP assay, T84 cells were grown in clear 24-well plates and pretreated for 20 minutes with 10 mM MgCl_2_ (or CaCl_2_) with or without 500 μM IBMX or vehicle control (0.2% DMSO). After that, cells were treated with 10 μM forskolin (for 5 min) and lysed by repeated freezing/thawing and centrifuged to remove cell debris. The supernatant was assayed for cAMP using the cAMP Parameter immunoassay kit (R&D Systems) according to the manufacturer’s instructions.

### Animals.

*Casr^fl/fl^* (strain 030647, C57BL/6 background), *Vil1-Cre* (strain 004586, C57BL/6 background), and WT mice (C57BL/6 or CD1 background) were obtained from The Jackson Laboratory. Intestinal epithelium–specific Casr-KO mice (*Vil1-Cre Casr^fl/fl^*) were generated by crossbreeding, and the genotype was confirmed by PCR. Animals were bred at the UCSF Laboratory Animal Resource Center, and experiments were conducted in adherence to the NIH *Guide for the Care and Use of Laboratory Animals* (National Academies Press, 2011). Both male and female mice were used in all experiments.

### Intestinal I_sc_ measurement in mice.

Jejunum was excised under anesthesia and soaked in an iso-osmolar solution containing 300 mM mannitol and 10 μM indomethacin. Mucosa was stripped from the serosa/muscle layers under a dissection microscope and mounted onto Ussing chambers containing Ringer’s solution on the basolateral side. For the apical side, a similar solution was used, except 120 mM NaCl was replaced with 60 mM NaCl and 60 mM sodium gluconate, and glucose was replaced with 10 mM mannitol. I_sc_ was measured as described above.

### Mouse models of cholera.

For closed-loop model, 8- to 12-week-old CD1 mice were fasted overnight with access to 5% dextrose in water but no solid food. Mice were anesthetized with isoflurane, and body temperature was maintained at 36°–38°C during surgery using a heating pad. After a small abdominal incision to expose the small intestine, mid-jejunal loops (2–3 cm in length) were isolated by sutures as described previously (50, 51). Loops were injected with 100 μL PBS (pH 7.4, 137 mM NaCl, 2.7 mM KCl, 8 mM Na_2_HPO_4_, 1.8 mM KH_2_PO_4_, 1 mM CaCl_2_, and 0.5 mM MgCl_2_) containing 1 μg cholera toxin or PBS alone. Some loops were injected with PBS with or without cholera toxin with 20 mM Mg^2+^. After loop injections, the abdominal incision was closed with sutures, and mice were allowed to recover from anesthesia. Intestinal loops were removed 3 hours after surgery, and the loop length and weight were measured to quantify fluid secretion. Loop fluid was aspirated with a syringe, and the Mg^2+^ concentration was quantified by colorimetric assay (MilliporeSigma).

For the intestinal perfusion model, 10- to 14-week-old C57BL/6 mice were fasted overnight. Under isoflurane anesthesia, the proximal duodenum and terminal ileum were cannulated. The small intestine was lavaged gently with saline (at 37°C) to clear the luminal contents. After draining the intestine, the ileal catheter was clamped, and cholera toxin (10 μg in 2 mL saline) or the saline control was instilled and dispersed through the small intestine. After 2 hours, the clamps were opened, and whole-gut perfusion was initiated at 0.2 mL/min with Ringer’s solution containing 2 mM ferrocyanide [Fe(CN)_6_, nonabsorbable volume marker] and 1 or 10 mM MgCl_2_ (in separate mice). After equilibration for 60 minutes, effluent samples were collected from the ileal catheter. The ferrocyanide concentration was determined in the infusate (ferrocyanide_i_) and effluent (ferrocyanide_e_) via absorbance, and net fluid transport (μL/min/cm) was calculated using the following previously described formula (53): perfusion rate: [perfusion rate × (ferrocyanide_i_)/(ferrocyanide_e_)]/length of gut (cm) × 1,000. In some experiments, we used the WHO ORS (TRIORAL, Trifecta Pharmaceuticals; 75 mM Na^+^, 65 mM Cl^–^, 75 mM glucose, 20 mM K^+^, 10 mM citrate) with and without 10 mM MgCl_2_.

### Statistics.

Experiments with 2 groups were analyzed using a 2-tailed, unpaired Student’s *t* test. For 3 or more groups, analysis was performed with 1-way ANOVA and a post hoc Newman-Keuls multiple-comparison test. In all analyses, a *P* value of less than 0.05 was considered statistically significant. Data indicate the mean ± SEM.

### Study approval.

The experimental protocols were approved by the IACUC of UCSF.

### Data availability.

Values for all data points in graphs are available in the Supplemental Supporting Data Values file.

## Author contributions

OC made the original discovery, conceptualized the study, and designed the experiments; LDSG, TC, RM, PDC, QG, and OC performed the experiments. LDSG, TC, and OC analyzed the data. OC obtained funding, supervised the study, and wrote the manuscript. LDSG and OC revised the manuscript. All authors read the manuscript and agreed to the submitted form. The order of the co–first authors’ names was determined alphabetically.

## Supplementary Material

Supplemental data

Supporting data values

## Version 1 11/14/2023

In-Press Preview

## Version 2 01/16/2024

Electronic publication
