# Supplementary material for: Mg2+ supplementation treats secretory diarrhea in mice by activating calcium-sensing receptor in intestinal epithelial cells
Source: J Clin Invest. 2024 Jan 16;134(2):e171249. doi: 10.1172/JCI171249 (PMC10786700; doi:10.1172/JCI171249)
Supplement: Supplemental data [file jci-134-171249-s216.pdf]

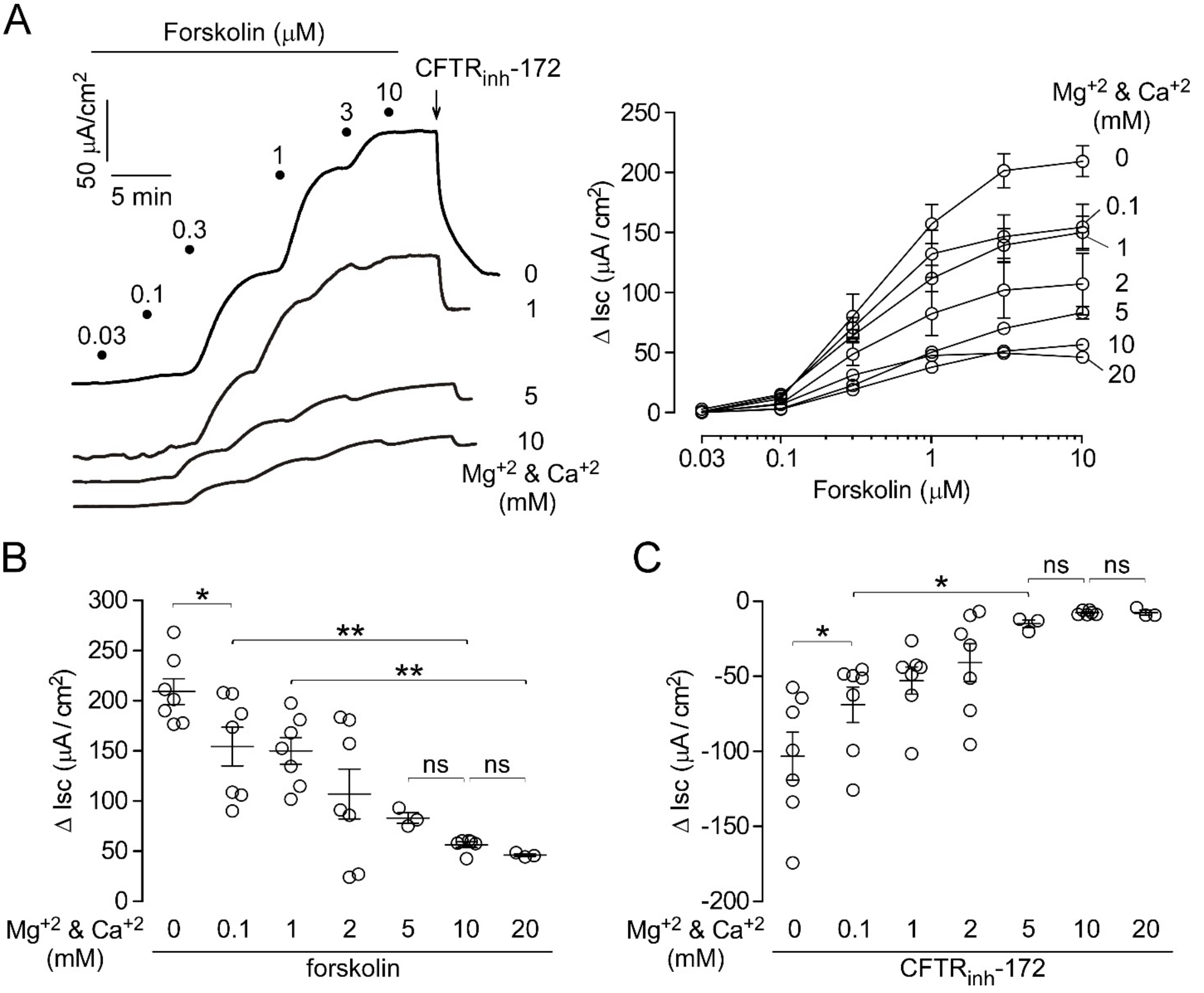

Supplementary Figure 1. Effects of simultaneously altered extracellular Ca^{2+} and Mg^{2+}concentrations on forskolin-induced I_{sc} in T84 cells. (A) Short-circuit current (I_{sc}) traces in T84 cells showing forskolin concentration response and $CFTR_{inh}$-172 (10 μM) inhibition following 20 min pretreatment with indicated concentrations of $MgCl_2$ and $CaCl_2$ *(left)*. Summary of changes in I_{sc} (ΔI_{sc}) from experiments *(right)*. **(B)** ΔI_{sc} induced by forskolin in the presence of different Mg^{2+} and Ca^{2+} concentrations. **(C)** ΔI_{sc} induced by $CFTR_{inh}$-172 at in the presence of different Mg^{2+} and Ca^{2+} concentrations. Mean ± S.E.M., n=3-5 per group, one-way ANOVA with Newman-Keuls multiple comparisons test, *$p<0.05$, **$p<0.01$, ns: not significant.

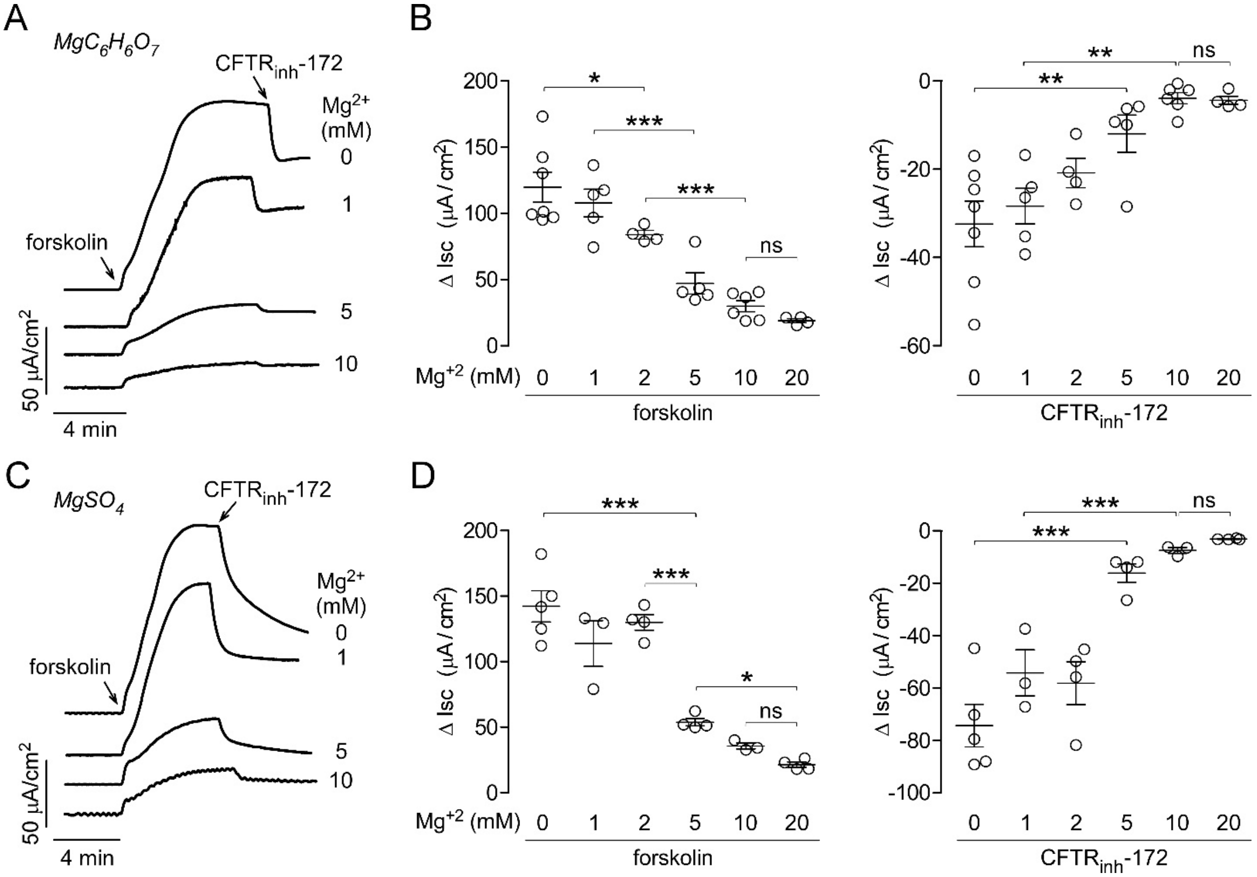

Supplementary Figure 2. Magnesium citrate and magnesium sulfate inhibit forskolin-induced I_{sc} in T84 cells. (A) Short-circuit current (I_{sc}) traces showing responses to maximal forskolin (10 μM) and $CFTR_{inh}$-172 (10 μM) inhibition following 20 min pretreatment with indicated concentrations of magnesium citrate ($MgC_6H_6O_7$). **(B)** Summary of I_{sc} changes (Δ I_{sc}) induced by forskolin (*left*) and $CFTR_{inh}$-172 (right) at different concentrations of $MgC_6H_6O_7$. Δ I_{SC} induced by CFTRinh-172 at different concentrations of $MgC_6H_6O_7$ *(right)*. **(C)** Short-circuit current (I_{sc}) traces showing responses to maximal forskolin (10 μM) and $CFTR_{inh}$-172 (10 μM) inhibition following 20 min pretreatment with indicated concentrations of magnesium sulfate ($MgSO_4$). **(D)** Summary of I_{sc} changes (Δ I_{sc}) induced by forskolin (*left*) and $CFTR_{inh}$-172 (right) at different concentrations of $MgSO_4$. Mean ± S.E.M., n=3-7 per group, one-way ANOVA with Newman-Keuls multiple comparisons test, *p<0.05, **p<0.01, ***p<0.001, ns: not significant.

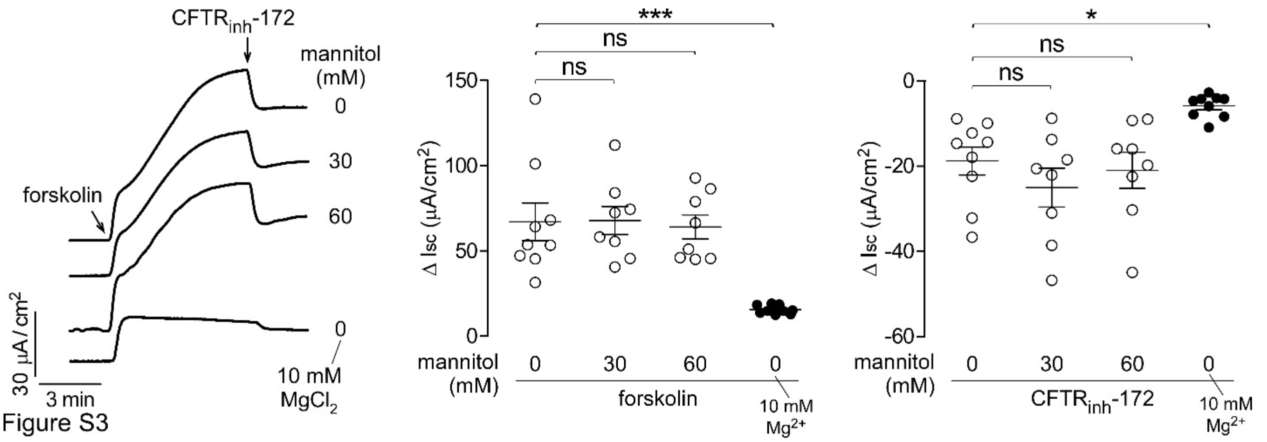

Supplementary Figure 3. Effects of solution osmolality on forskolin-induced I_{sc} in T84 cells. (*left*) Short-circuit current (I_{sc}) traces showing responses to maximal forskolin (10 μM) and CFTR$_{inh}$-172 (10 μM) inhibition following 20 min pretreatment with indicated concentrations of mannitol. Summary of I_{sc} changes (ΔI_{sc}) induced by forskolin (*center*) and CFTR$_{inh}$-172 (*right*) at different concentrations of mannitol. In some experiments, cells were treated with 10 mM $MgCl_2$ in the absence of mannitol. Mean ± S.E.M., n=8-9 per group, one-way ANOVA with Newman-Keuls multiple comparisons test, *$p<0.05$, ***$p<0.001$, ns: not significant.

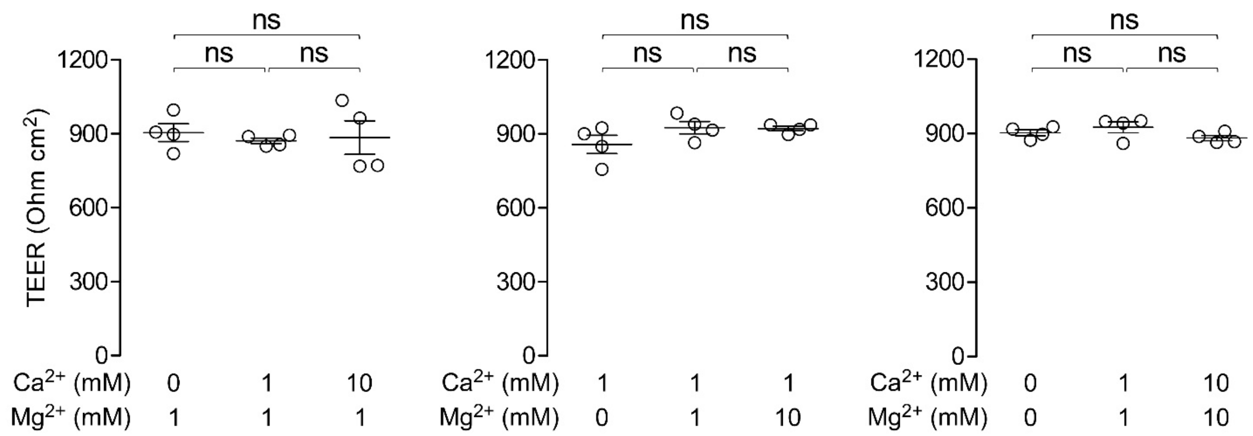

Figure S4

Supplementary Figure 4. Effects of Ca^{2+} and Mg^{2+} concentrations on barrier permeability in T84 cells. Transepithelial electrical resistance (TEER) measurements in T84 cells after 60 min bathing in Ringer solution with indicated concentrations of $CaCl_2$ and $MgCl_2$. Mean ± S.E.M., n=4 per group, one-way ANOVA with Newman-Keuls multiple comparisons test, ns: not significant.

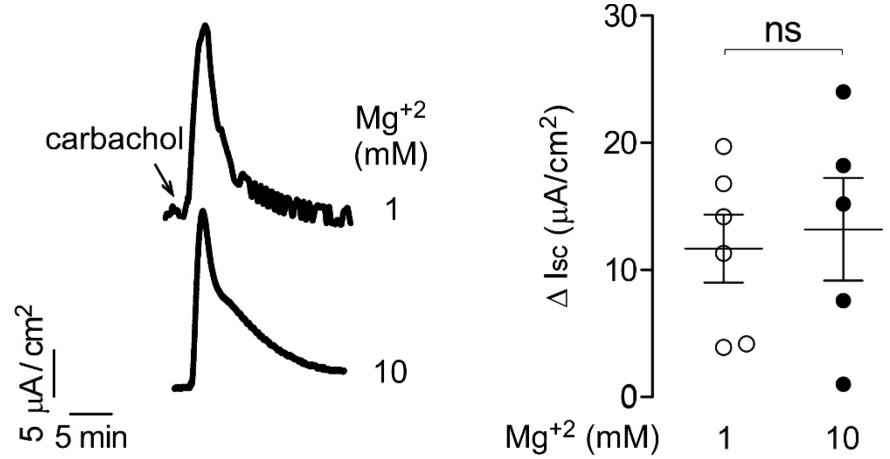

Figure S5

Supplementary Figure 5. Extracellular Mg^{2+} concentration does not affect carbachol-induced secretory currents in T84 cells. (*left*) Short-circuit current (I_{sc}) traces showing responses to carbachol (100 μM) following 20 min pretreatment with indicated concentrations of $MgCl_2$. (*right*) Summary of I_{sc} changes (Δ I_{sc}) induced by carbachol. Mean ± S.E.M., n=5-6 per group, Student's t-test, ns: not significant.
